# Lung adenocarcinoma with thyroid metastasis: a case report

**DOI:** 10.1186/s13104-017-2449-4

**Published:** 2017-03-21

**Authors:** A. Dao, H. Jabir, A. Taleb, N. Benchakroun, Z. Bouchbika, T. Nezha, H. Jouhadi, S. Sahraoui, A. Benider

**Affiliations:** 1 Centre Mohammed VI pour le Traitement des Cancers, CHU Ibn Rochd, Casablanca, Morocco; 2 Centre Hospitalier Universitaire Yalgado Ouedraogo, Ouagadougou, Burkina Faso

**Keywords:** Lung, Thyroid, Metastasis, Surgery, Chemotherapy

## Abstract

**Background:**

The metastases of a primary lung cancer over the thyroid gland are extremely rare. We report on an unusual presentation of thyroid metastasis of lung cancer in order to improve the management of similar cases.

**Case presentation:**

Three years ago, a Moroccan male 59-year-old was admitted for dyspnea, dry cough, and chest pain. He had smoked about 30 cigarette packs a year. Clinical examination revealed a right thyroid nodule. Chest and neck computed tomography (CT) scan showed a proximal left tumor in contact with the pulmonary artery and revealed a suspected nodule in the right lobe of the thyroid with homolateral neck node. Transbronchial biopsy was performed and pathological examination revealed adenocarcinoma of the lung and positive for thyroid transcription factor. Other explorations carried out, such as brain CT, bone scan and abdominal ultrasound were normal. After a repeated negative fine needle aspiration biopsy of the suspected nodule of the right lobe of the thyroid, we performed total thyroidectomy with neck dissection. An anatomopathologic exam revealed a tubulopapillary adenocarcinoma poorly differentiated. An Immunohistochemistry showed positive tumor cells with TTF1 and cytokeratin (CK) 7 but negative cells with thyroglobulin and CK20. Thus, the pulmonary tumor was classified stage IV. Chemotherapy based on the combination of cisplatin and etoposide was conducted along with supportive care. The tumor grew up with brain metastases after three cycles of chemotherapy. Unfortunately, the patient died 2 months after despite brain radiotherapy.

**Conclusion:**

We presented a medical case of a patient with thyroid metastasis resulting from a pulmonary adenocarcinoma which has rapidly evolved to brain metastases. The prognosis was pejorative in our clinical case (5 months after admission).

## Background

The metastases of a primary lung cancer to the thyroid gland are extremely rare [1]. The differential diagnosis with primary cancer of the thyroid is difficult because of the non-specific clinical symptoms and imaging [2, 3]. Malignant tumors of the thyroid can be subdivided into two groups: the primary tumors which have a slow evolution, a usually locoregional extension and a good prognosis. The histological types of these primary tumors are papillary carcinomas, follicular carcinomas, anaplastic carcinomas, medullary carcinomas and lymphoma. The second group consists of intrathyroid metastases. These metastases account for 2 to 4% of all clinical cases with thyroid malignant tumors [3, 4]. The prognosis is generally poor and depends on the histologic type of the primary tumor. The most commonly primary cancers implicated are kidney cancers, breast cancers, lung cancers, gastrointestinal cancers, followed by melanomas and lymphomas [5]. The proportion of these intrathyroid metastases reaches 24.2% in autopsy series, indicating that such metastatic involvements are much more common than has been clinically appreciated [3]. In these autopsy series, the breast and lung cancers are the predominant etiologies in North America [6] and digestive cancers (esophagus and stomach) in Asia [7]. In these regions, pulmonary origin is, respectively, 13.6 [6] and 25% [7] of the intrathyroid metastases. Other authors [2] noted that pulmonary origin represented 45.4% of intrathyroid metastases. There were five cases of thyroid metastases from pulmonary among 11 cases reported in all. The histological types of these lung cancers were squamous cell carcinomas (two cases), non-small cell lung cancer (two cases) and anaplastic small cell carcinoma (1 case). The intrathyroid metastases from a lung adenocarcinoma is not commonly reported particularly in male patient. [2]. Only few cases of intrathyroid metastases from primary lung adenocarcinoma were reported in literature [2]. In general, lung adenocarcinoma induces the distant metastases in the liver, adrenal, bone and brain. The intrathyroid metastases from pulmonary origin have been not yet reported in the Maghreb region. Recently, we experienced a case of lung adenocarcinoma with thyroid metastasis. The purpose of this report is to present this case and discuss the diagnostic difficulties of unexpected or unusual presentations of thyroid metastasis of a lung adenocarcinoma.

## Case presentation

**Fig. 1:**
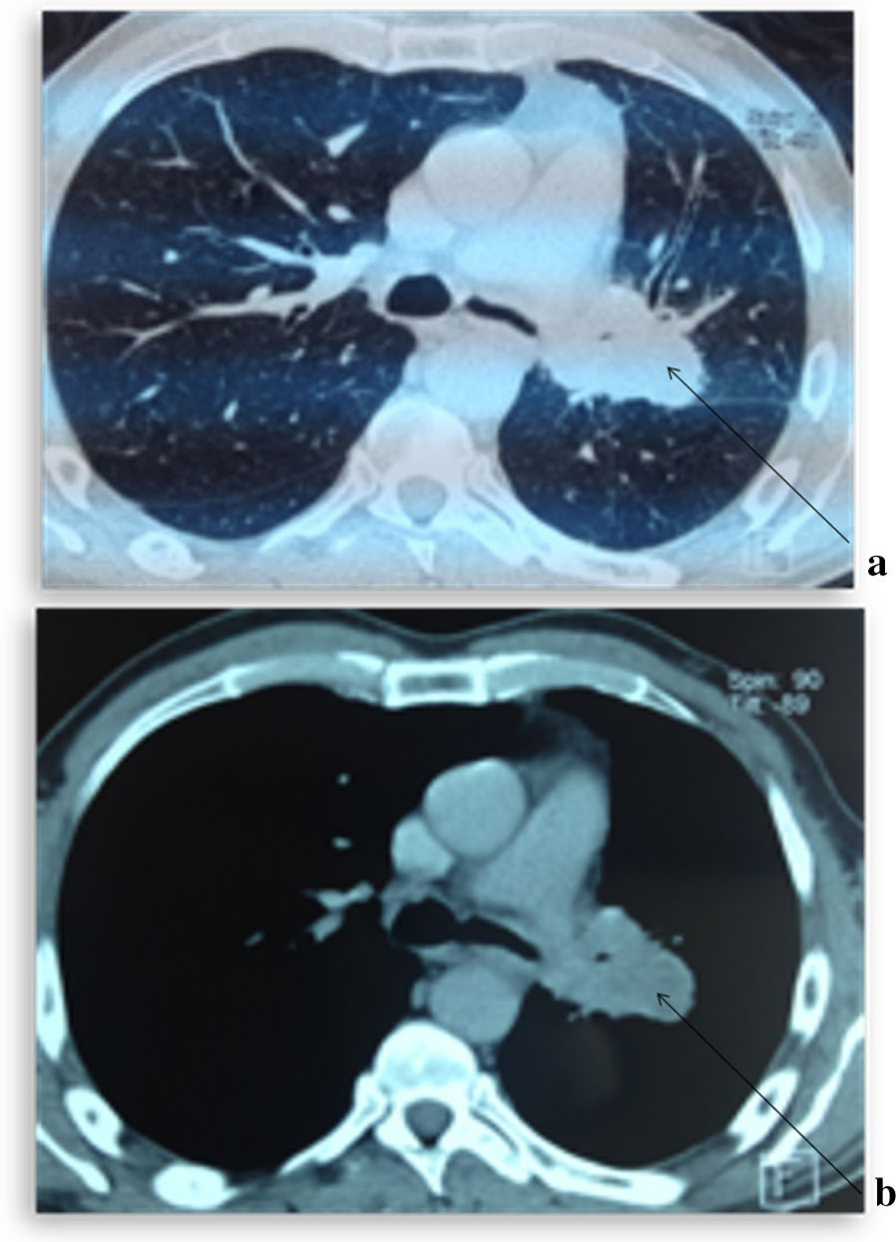
Computed tomography of the chest in pulmonary side (**a**) and mediastinal side (**b**). Helical chest CT scan. Axial cut after contrast injection. The pulmonary window (**a**) showed a heterogeneous tissue process developed at the expense of the posterior segment of the left upper lobe spiculated contours with lymphangitis around the tumor. This tumor measuring 5.5 × 6 cm. On mediastinal window (**b**): there were no pathologic mediastinal lymph nodes. Vascular axes were free and had normal caliber

**Fig. 2:**
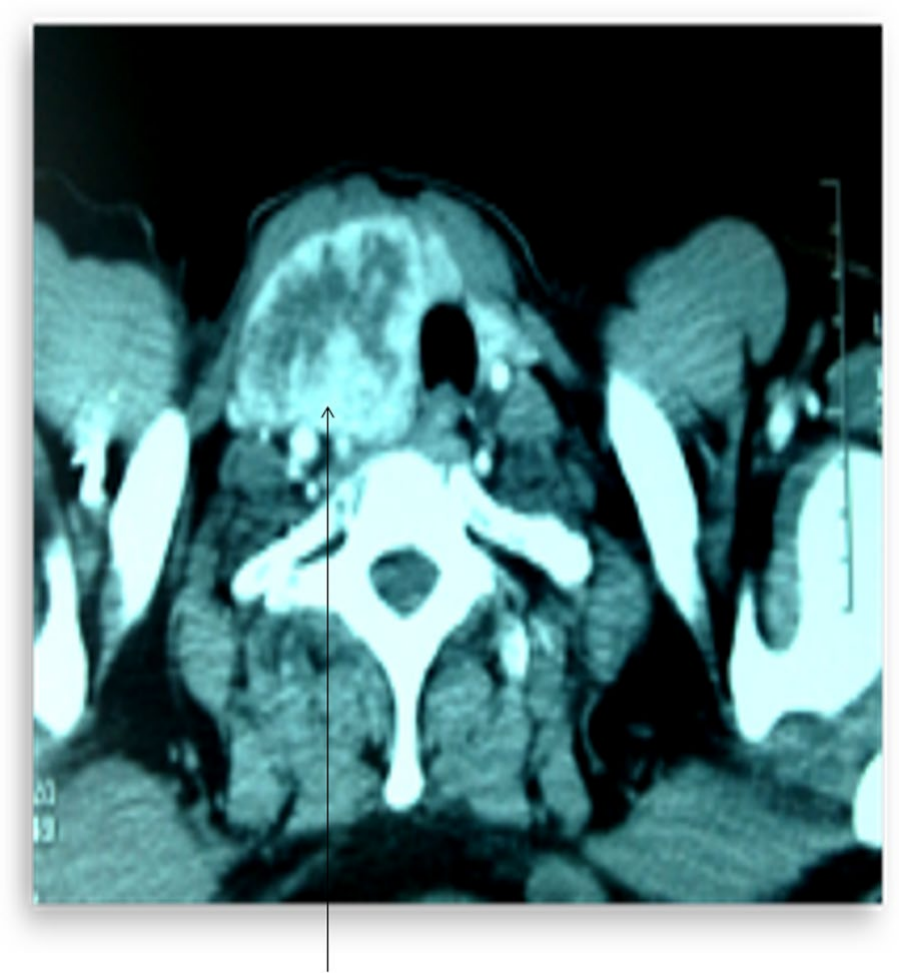
Cervical computed tomography scan—axial cup showing a large nodule in right lobe of thyroid, heterodense, dipping into the upper mediastinum and measuring 8 × 4 cm

Three years ago, a 59-year-old male Moroccan presented to the hospital with a dyspnea, dry cough, and a chest pain that had started 6 months before. He had smoked about 30 cigarette packs a year. He was diagnosed non-insulin dependent diabetes 1 year before and was under oral anti-diabetics. He had undergone a successfully surgical intervention for priapism 4 years before the current episode of sickness. His sister is known to have a breast adenocarcinoma. Physical examination at admission revealed a patient with performance status according to World Health Organization (WHO) quote one, and dyspnea type II according to NYHA (New York Heart Association). The pulmonary examination was poor. Head and neck examination found a right thyroid nodule without clinical signs of hypothyroidism or hyperthyroidism. Chest and neck computed tomography (CT) scan were performed and revealed a tumor in the left upper lobe of the lung, in contact with the pulmonary artery (Fig. 1a: chest axial cup CT-scan pulmonary window). The pulmonary tumor measured 5.5 × 6 cm without mediastinal lymph nodes (Fig. 1b: chest axial cup CT-scan mediastinal window). The neck CT-scan showed one nodule in the right lobe of the thyroid plunging into the superior mediastinum measuring 8 × 4 cm with homolateral cervical lymph nodes (Fig. 2). The level of the thyroid stimulating hormone (TSH) was normal. For the pulmonary tumor, we performed transbronchial biopsies. Pathological examination of the biopsy revealed an adenocarcinoma of the lung, positive thyroid transcription factor (TTF-1).

**Fig. 3:**
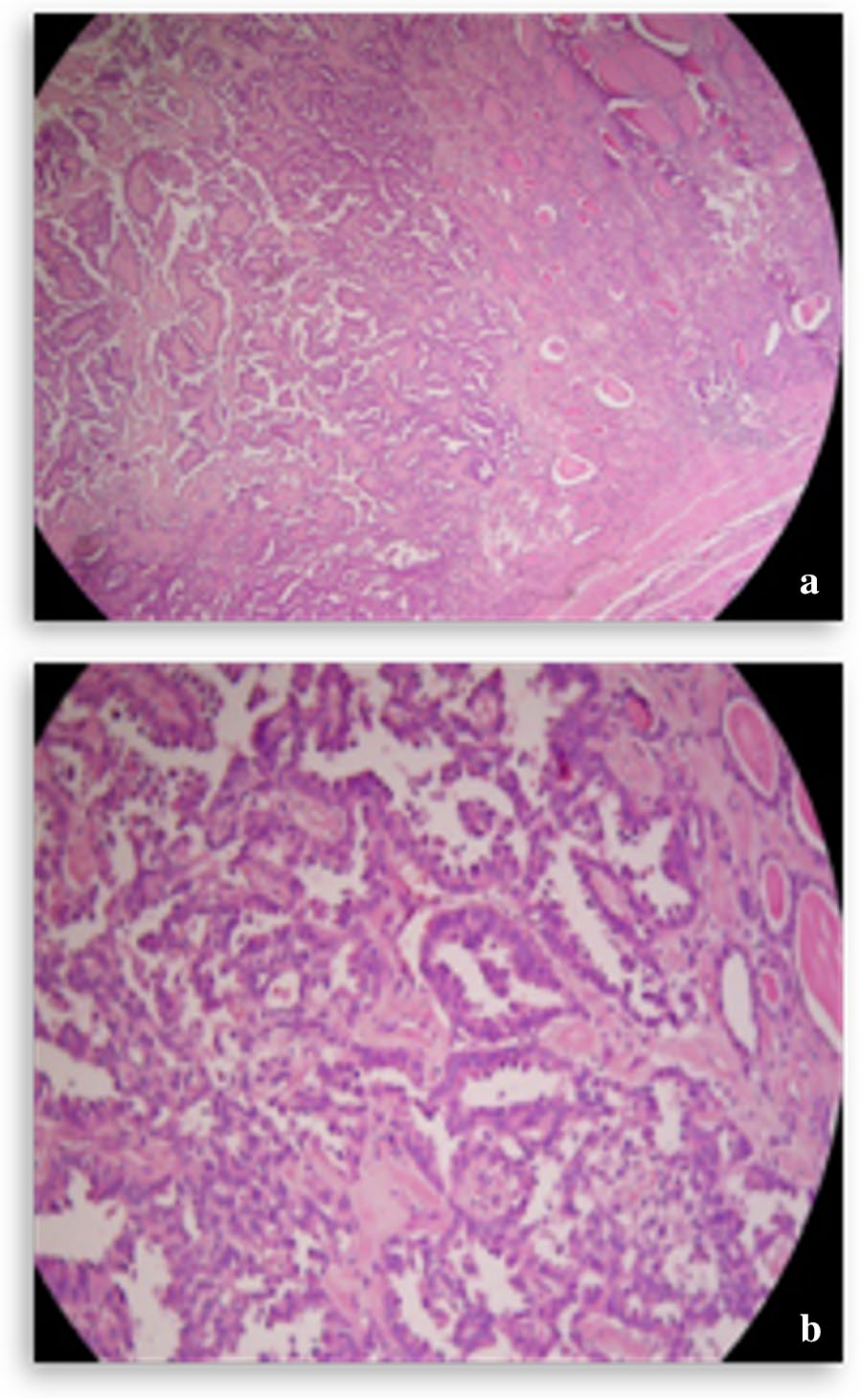
Pathological examination of the thyroid tumor showing **a** tubulo-papillary moderately differentiated adenocarcinoma (×200), **b** strong swelling

**Fig. 4:**
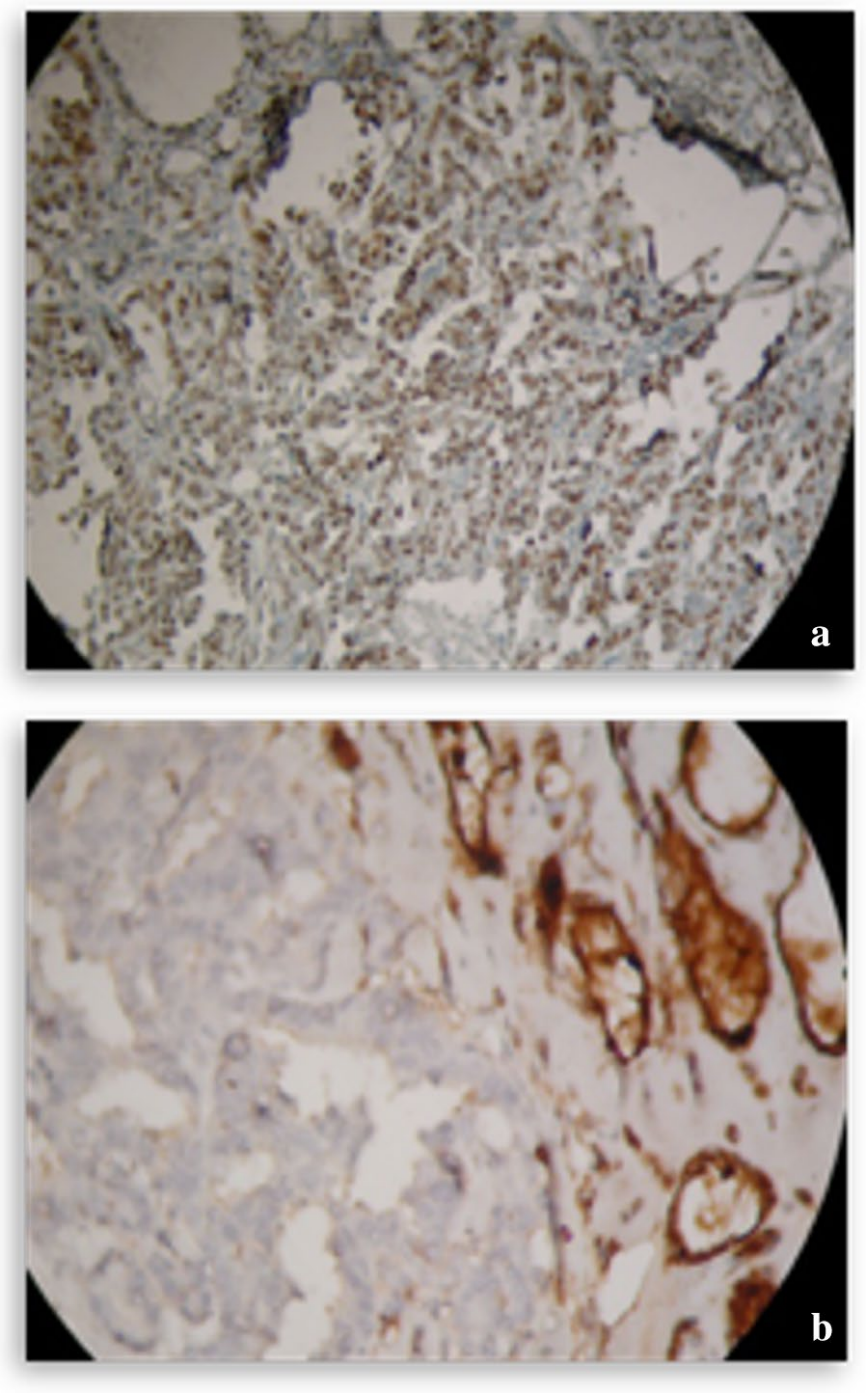
Immunohistochemistry of the thyroid tumor showing **a** diffusely positive cells for TTF1 and CK7 (×200), **b** negative cells for thyroglobulin and CK20 (×200)

Other explorations conducted such as brain CT and bone scan and abdominal ultrasound were normal. We concluded a diagnosis of pulmonary adenocarcinoma classified T4N0M0 with a suspected nodule of the thyroid right lobe. We performed a repeated fine needle aspiration biopsy (FNAB) of the thyroid right lobe but the anatomopathologic results were negatives. We decided to performed a total thyroidectomy and lymph node dissection after extemporaneous anatomopathologic exam, confirming the malignancy of the thyroid right lobe nodule. The pathologic examination revealed a moderately differentiated tubulopapillary adenocarcinoma (Fig. 3a, b) and three lymph nodes with extracapsular effraction. The immunohistochemistry showed positive tumor cells with TTF1 and cytokeratin (CK) 7 (Fig. 4a) but negative tumor cells with thyroglobulin and CK20 (Fig. 4b). Thus, the pulmonary tumor was classified stage IV (T4N0M1) according to the 2009 UICC (Union for International Cancer Control) staging. The multidisciplinary team found that lung tumor was inoperable because it was in contact with the pulmonary artery. The decision to administrate chemotherapy followed by the chemoradiation was retained because there was lack of places on the linear accelerator. A chemotherapy based on the combination of cisplatin and etoposide was administrated along with supportive care. The chemotherapy consisted of the combination of cisplatin and etoposide that were administered according to the following schema: for every cycle, cisplatin 75 mg/m^2^ on day 1 and etoposide 100 mg/m^2^ on day 1–3. The length of time between the treatments was 21 days. In total, two cycles of this chemotherapy were performed. We didn’t report any side effect regarding this chemotherapy. After the two cycles, the patient presented dizziness and headaches. A brain magnetic resonance imaging (MRI) was performed and showed multiples brain metastases. The cranial palliative radiotherapy 10 × 3 grays including five fractions per week was realized with best supportive care. This radiation therapy was well tolerated. Particularly, there was no deleterious effect on cognition. After the radiation therapy, we decided to go on with the chemotherapy administration. But the performance status of the patient was poor. Unfortunately, despite reanimation measures and best supportive care, the patient died 2 months later after brain radiotherapy.

## Discussion

Despite the complexity of the clinical symptomatology and our work context marked by our patients’ lack of financial means, we were able to make a single diagnosis. It is the result of systematic multidisciplinary approach of our working team. Unfortunately, the survival of our patient did not exceed 6 months despite the use of best therapeutic strategy. Brain metastases occurred earlier, 2 months after the start of the chemotherapy. Did they go undetected in the initial brain scan? Metastasis of the thyroid are synchronous or metachronous of primary neoplasm, arising from 1 month to 26 years (median 54 months) [4, 8]. In our case, the thyroid metastasis was synchronous. The clinical symptoms were not specific. In the majority of cases, the revelation mode is a unifocal lesion of the thyroid, although multifocal or diffuse involvement is also seen [7]. The biological thyroid is usually in favor of euthyroidism and the thyroglobulin is normal. Rare cases of thyroid dysfunction have been reported; it was hypothyroidism due to metastatic infiltration and the replacement of the thyroid by cancer. In addition, it was hyperthyroidism due to follicular destruction by metastasis cells [9, 10]. The controversy about the place of surgery and the extent of surgical resection of intrathyroid metastases is widely reported in the literature [4]. The decision of conducting surgery of intra thyroid metastase is based on histological type of primary cancer, the location of intrathyroid secondary lesions, the kinetics of primary tumor and metastatic extension type (oligo or poly metastatic) [2]. Many authors [2, 3, 8, 10–12] recommend the total thyroidectomy, especially when the rest of the workup proves negative. For those authors, the total thyroidectomy does not increase morbidity. It outweighs any secondary lesions intrathyroids multifocal and avoids morbidity associated with locoregional evolution or local recurrence potential. In our case, there was no other distant metastases. Some authors [5] do not agree with the total thyroidectomy because the rest of the thyroid would continue to produce hormones that could have cytostatic properties. Those authors recommend the thyroidectomy when the primary tumor grows rapidly or when there are cervical symptom regarding tracheal compression The histologic type of our case (adenocarcinoma) and the tumor location in lung could explain the rapid evolution of the intrathyroid metastasis.

Other authors have analyzed the prognostic impact of thyroidectomy for intrathyroïd metastases [7] and concluded that thyroidectomy prevents subsequent metastasis dissemination and lengthens the interval between the diagnosis of metastasis and the death of patients but doesn’t affect the overall survival. The total thyroidectomy performed on our patient could be considered excessive. Nevertheless, the prolonged survival (7–22 years) reported in intrathyroïd metastase of renal cell carcinoma by many authors after thyroid resection (loboisthmectomie or total thyroidectomy) [2] could be in favor of our approach. The performance of a PetScan could help eliminate the involvement of the rest of the thyroid. We did not have this PetScan during the management of this case. Mean Survival rate is 2–60 months (mean 19 months) [13] for all thyroid metastasis and about 2 months for metastasis of a lung primary cancer of the thyroid gland [11, 14]. The poor survival of our case report could be explained by the lack of knowledge of the status as regards the epidermal growth factor receptor (EGFR) and anaplastic lymphoma kinase (ALK) mutations. Indeed, the identification of prospective molecular abnormalities can help to define the therapeutic strategy. The search for these mutations is indicated in the following situations: non-small cell lung cancer including adenocarcinoma in advanced stage, female patient, no smoking or little smoking patient or Asian patient. Three mechanisms characterize these mutations in non-small cell lung cancers. An activating mutation of the genes of growth factor receptors (epidermal growth factor, human epidermal growth factor receptor-2) and intracellular transduction pathways: Kirsten rat sarcoma viral oncogene homolog (KRAS)/proto-oncogene B-Raf/dual threonine and tyrosine recognition kinase (MEK) and phosphatidylinositol-4,5-bisphosphate 3-kinase catalytic subunit alpha (PIK3CA)/protein kinase AKT/mammalian target of rapamycin (mTOR) pathway. These mutations stimulate tumor growth. Secondly, a translocation of the following genes: ALK, ROS proto-oncogene 1, receptor tyrosine kinase (ROS1) and The rearranged during transfection (RET) proto-oncogene. These mutations induce a cascade of signals promoting cell proliferation, recruitment of new vessels and the ability of cells to move in blood circulation. Thirdly, the amplifications of genes or overexpression of membrane proteins. In the EGFR and ALK mutations, targeted therapy inhibits the growth of tumor cells by blocking the activity of tyrosine kinase [15]. The EGFR and ALK status of our patient was not determined due to the absence of these tests in our structure and their high cost. In addition, targeted therapies in these mutations do not exist in our country presently and their costs are also unaffordable for our patients.

## Conclusion

We presented a medical case of a patient with thyroid metastasis from pulmonary adenocarcinoma which has rapidly evolved into the development of brain metastases. The total thyroidectomy and chemotherapy did not appear to have any impact on the course of disease progression. Two months after the starting of chemotherapy, the brain metastases appeared earlier despite the performance of a brain CT-scan in the initial workup. We recommend a brain MRI in the initial workup when there is suspected nodule of thyroid. The search of mutation of EGFR and ALK and administration of appropriate targeted therapy could improve survival of patients with advanced lung adenocarcinoma. The prognosis was pejorative in our clinical case (5 months after admission) and confirms the rapid progression to death, reported in the literature as regards lung adenocarcinoma with intrathyroid metastasis.

## Abbreviations

TTF-1: thyroid transcription factor
CT-scan: computed tomography scan
CK: cytokeratin
MRI: magnetic resonance imaging
EGFR: epidermal growth factor receptor
ALK: anaplastic lymphoma kinase
